# Supplementary material for: Modulation of Early Mitotic Inhibitor 1 (EMI1) depletion on the sensitivity of PARP inhibitors in BRCA1 mutated triple-negative breast cancer cells
Source: PLoS One. 2021 Jan 7;16(1):e0235025. doi: 10.1371/journal.pone.0235025 (PMC7790533; doi:10.1371/journal.pone.0235025)
Supplement: S1 Table — (DOCX) [file pone.0235025.s001.docx]

**Supplementary Table 1**

| siRNA | sequence | source |
| --- | --- | --- |
| EMI1 si1 against CDS | CGAAGUGUCUCUGUAAUUA | IDT |
| EMI1 si2 against 3`UTR | UUACUGAUCAUGAAUGUUA | IDT |
| control si targets luciferase | AACGUACGCGGAAUACUUCGA | IDT |
