# Supplementary material for: Modulation of Early Mitotic Inhibitor 1 (EMI1) depletion on the sensitivity of PARP inhibitors in BRCA1 mutated triple-negative breast cancer cells
Source: PLoS One. 2021 Jan 7;16(1):e0235025. doi: 10.1371/journal.pone.0235025 (PMC7790533; doi:10.1371/journal.pone.0235025)
Supplement: S2 Table — (DOCX) [file pone.0235025.s002.docx]

**Supplementary Table 2**

| **drug** | **source** | **identifier** |
| --- | --- | --- |
| Olaparib | AdooQ | Cat. No A10111-10 |
| talazoparib (BMN-673) | AdooQ | Cat. No. A11243 |
| CHK1 inhibitor (SB218078) | AdooQ | Cat. No. A1548 |
| Cisplatin | Enzo | Cat. No. ALX-400-040-M050 |
| MEK inhibitor selumetinib AZD6244 | AdooQ | Cat. No. A10257 |
