# Supplementary figures and images for: Modulation of Early Mitotic Inhibitor 1 (EMI1) depletion on the sensitivity of PARP inhibitors in BRCA1 mutated triple-negative breast cancer cells
Source: PLoS One. 2021 Jan 7;16(1):e0235025. doi: 10.1371/journal.pone.0235025 (PMC7790533; doi:10.1371/journal.pone.0235025)

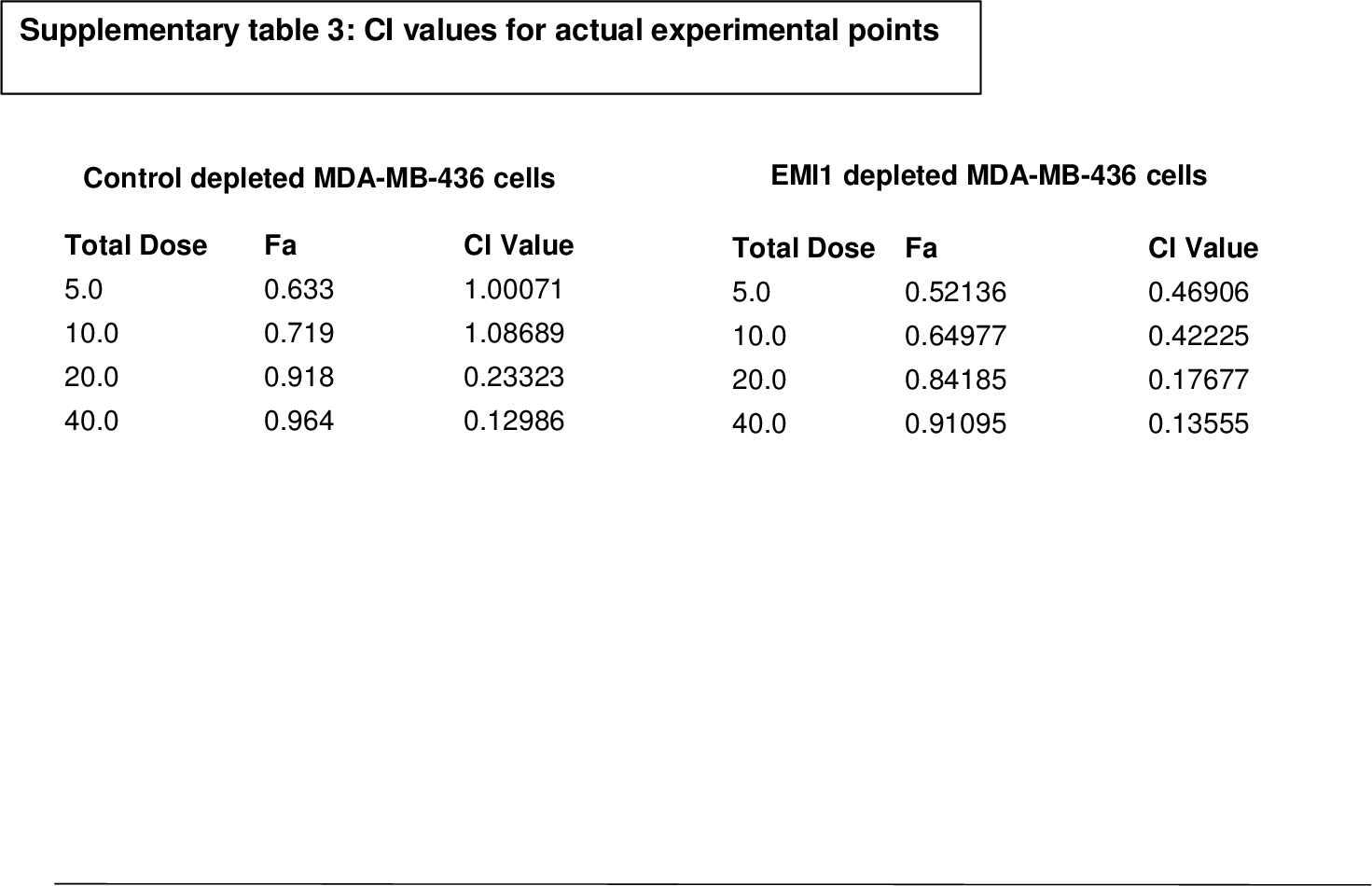

Supplement: S3 Table — A constant-ratio drug combinations 1:1 of olaparib:CHK1 I was analyzed in all cells tested. When analyzing the proliferation of EMI1-depleted cells versus control siRNA transfected cells, the CI values for all concentrations tested were lower than 1, suggestive of a synergistic effect between the two drugs under these conditions. (TIF) [file pone.0235025.s003.tif]
