# Supplementary material for: Modulation of Early Mitotic Inhibitor 1 (EMI1) depletion on the sensitivity of PARP inhibitors in BRCA1 mutated triple-negative breast cancer cells
Source: PLoS One. 2021 Jan 7;16(1):e0235025. doi: 10.1371/journal.pone.0235025 (PMC7790533; doi:10.1371/journal.pone.0235025)
Supplement: S1 File — (PDF) [file pone.0235025.s004.pdf]

Figure 1 B left

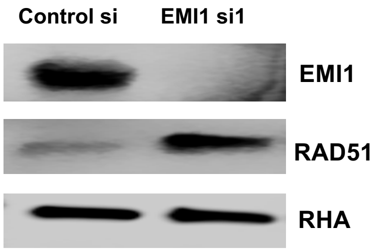

Cropped

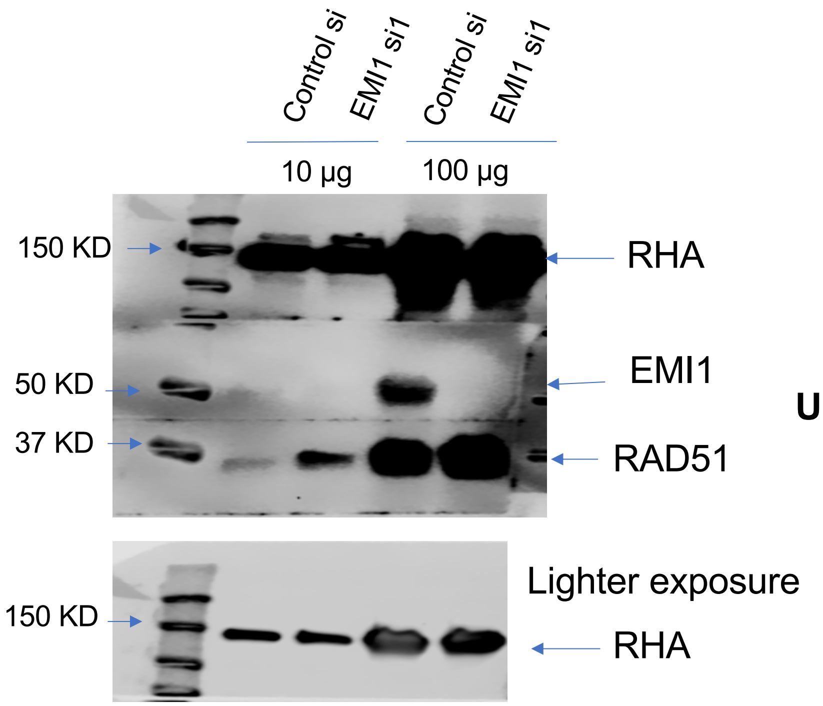

Uncropped

Figure 1 B Right

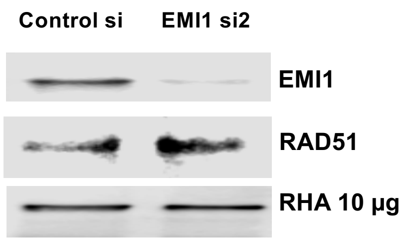

Cropped

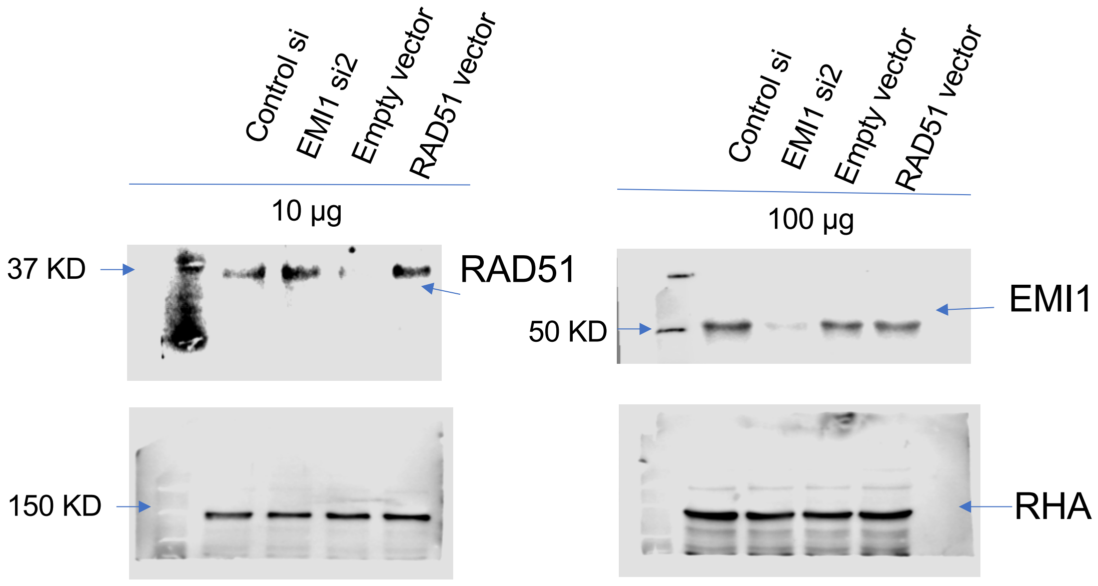

Uncropped

Figure 1 C Left

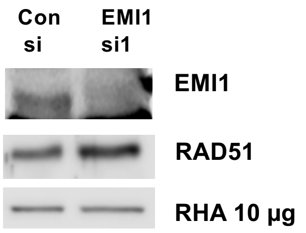

Cropped

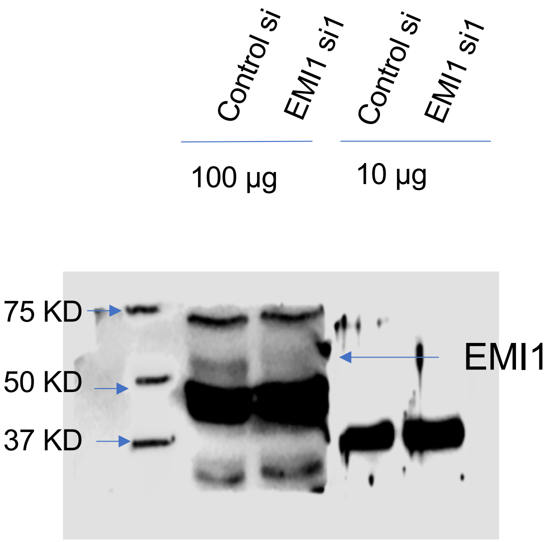

Lighter exposure

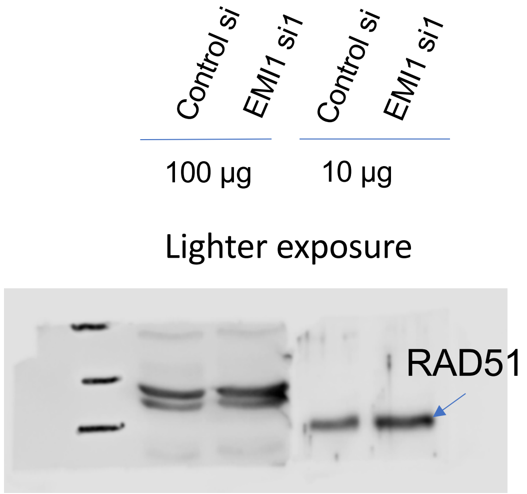

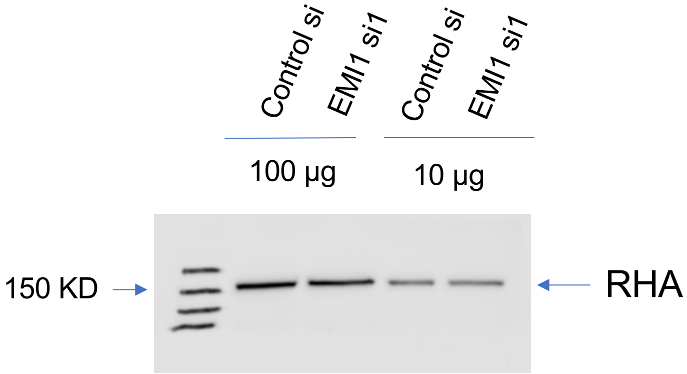

Uncropped

Figure 1 C Right

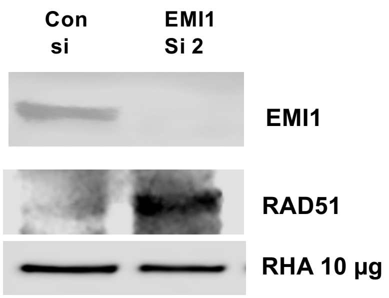

Cropped

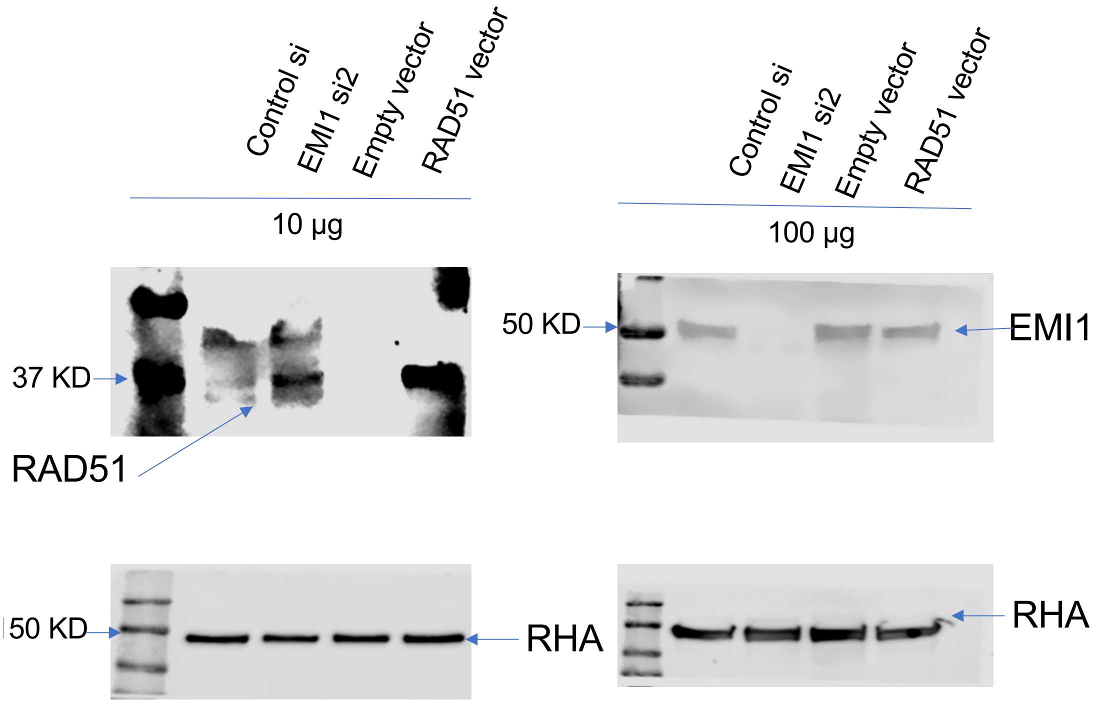

Uncropped

Figure 2 A left

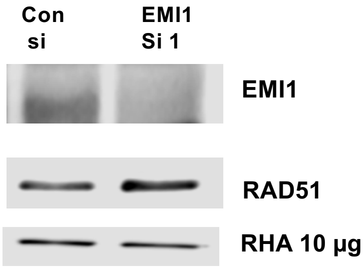

Cropped (MDA-MB-436 cells)

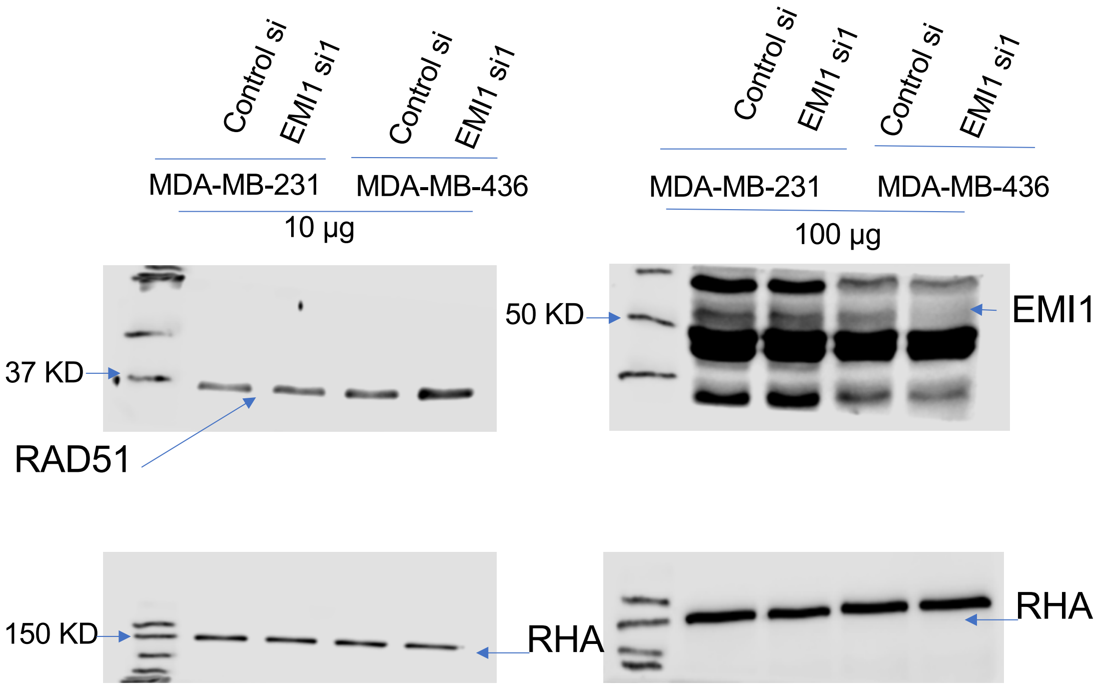

Uncropped

Figure 2 A Right

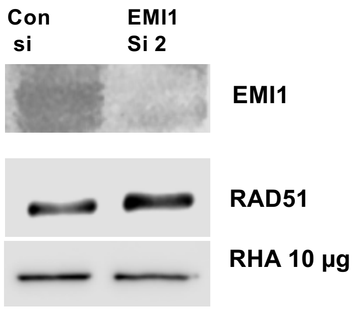

Cropped (MDA-MB-436 cells)

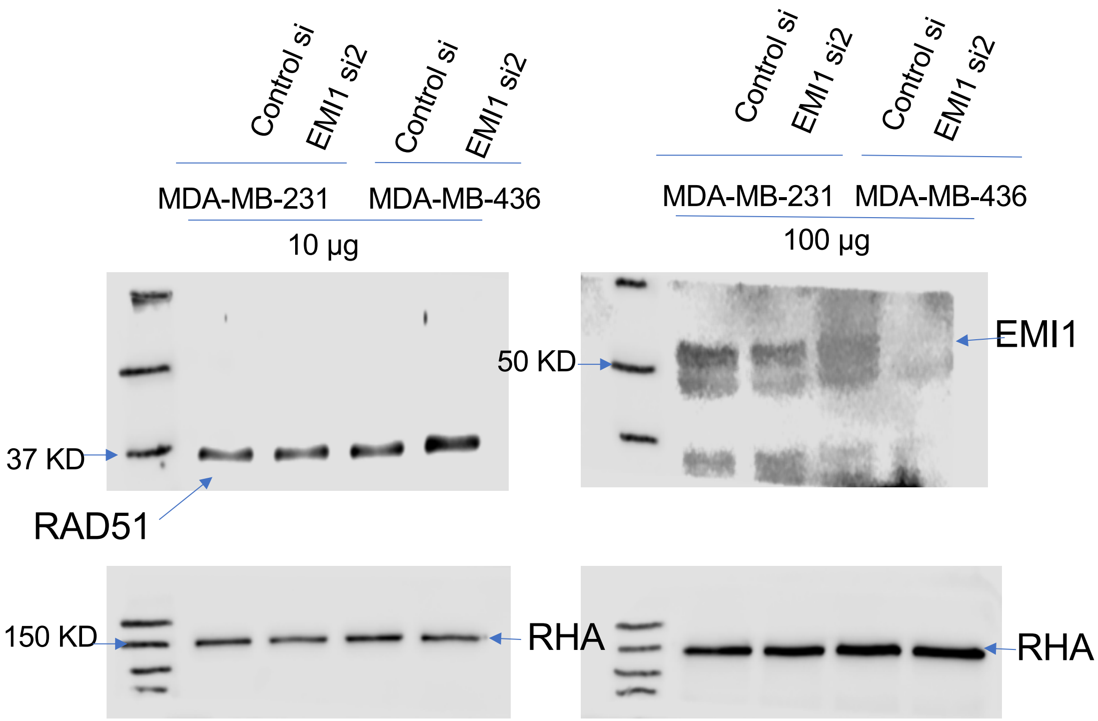

Uncropped

Figure 2 B left

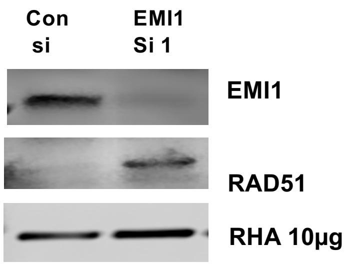

Cropped

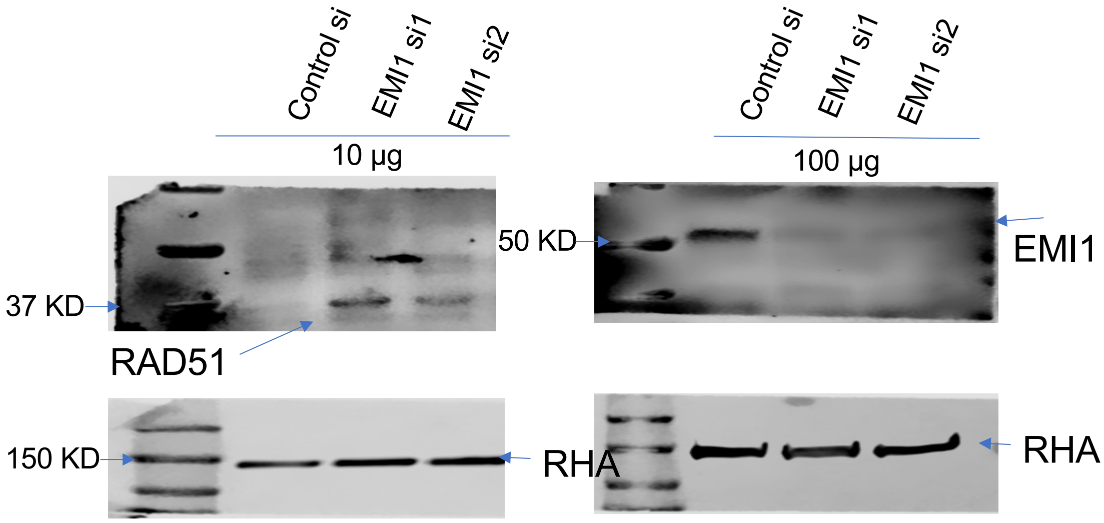

Uncropped

Figure 2 B Right

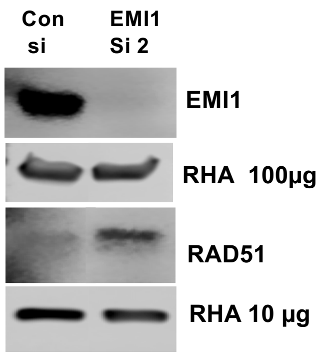

Cropped

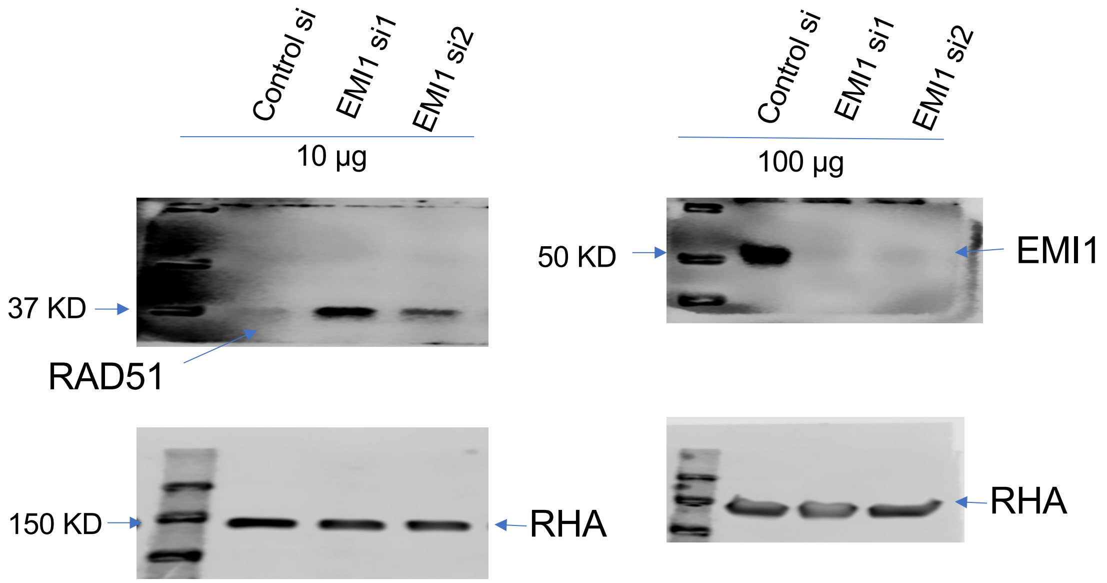

Uncropped

Figure 2 C

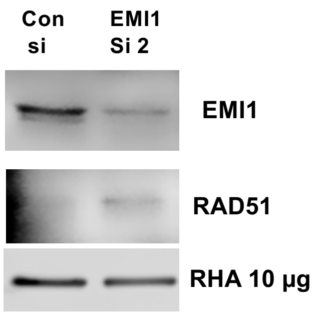

Cropped

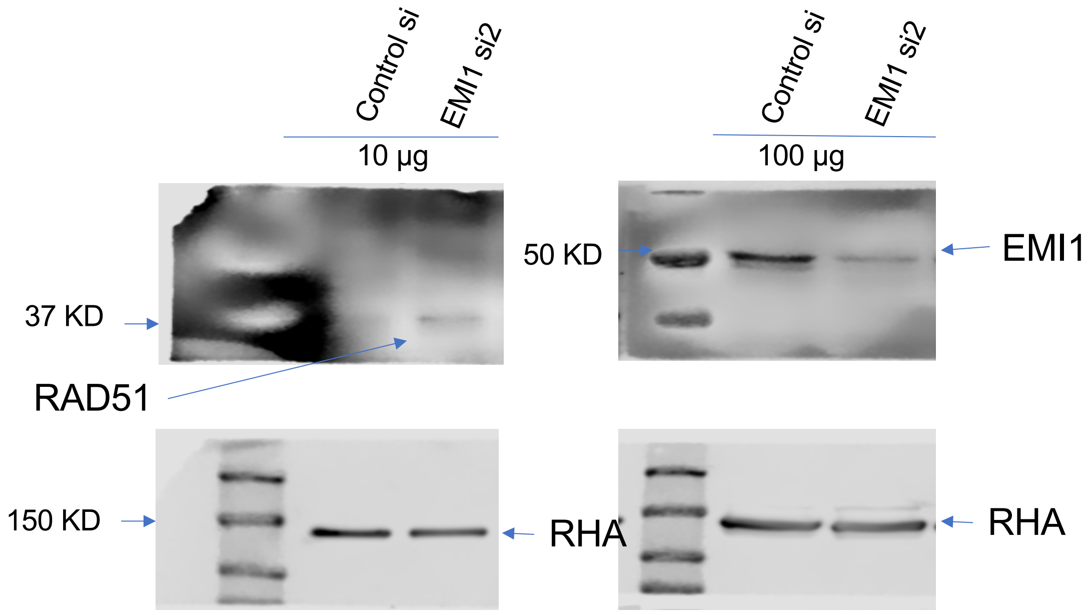

Uncropped

Figure 3 C

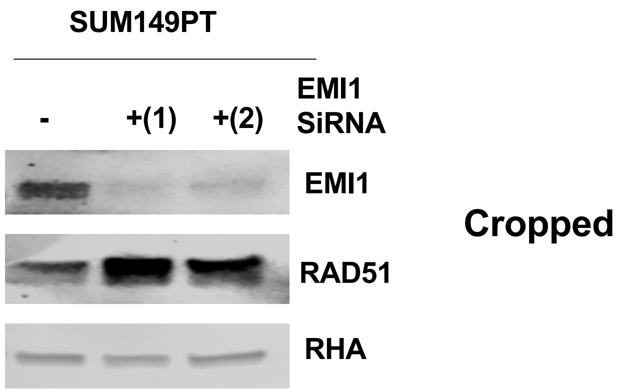

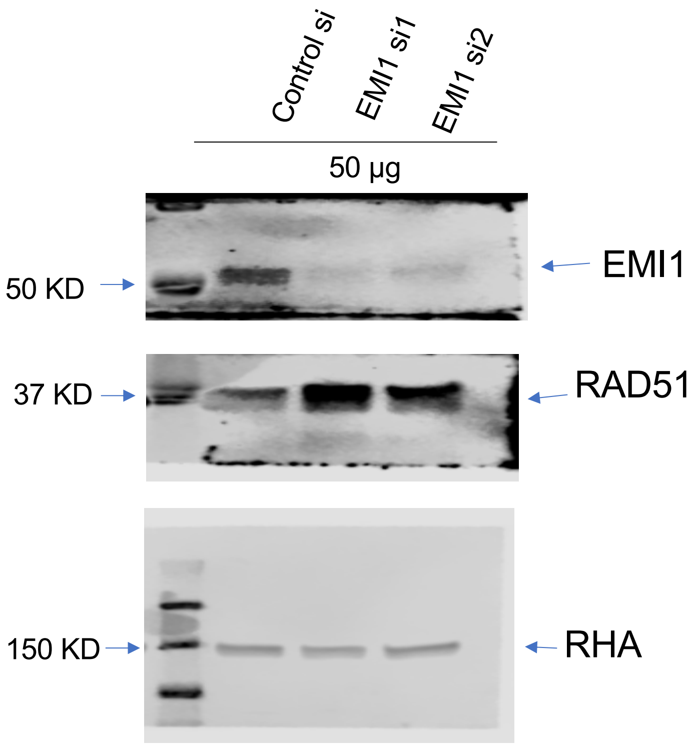

Uncropped

Figure 4 A

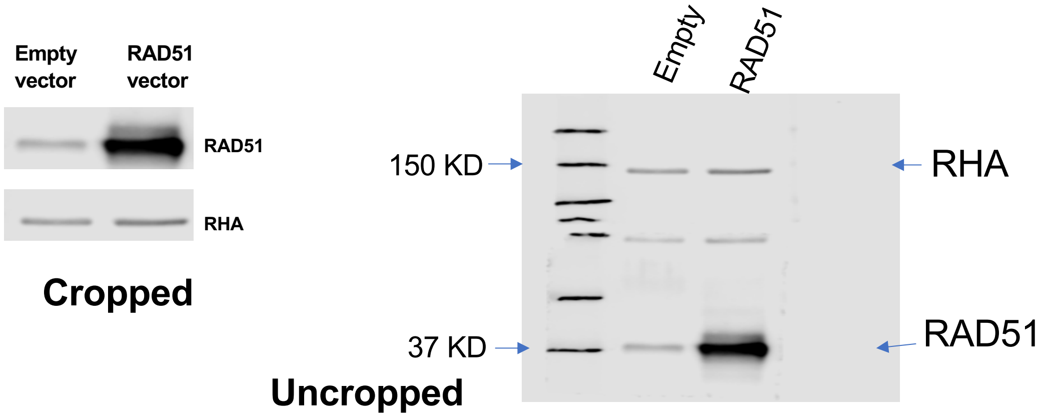

Figure 4 B

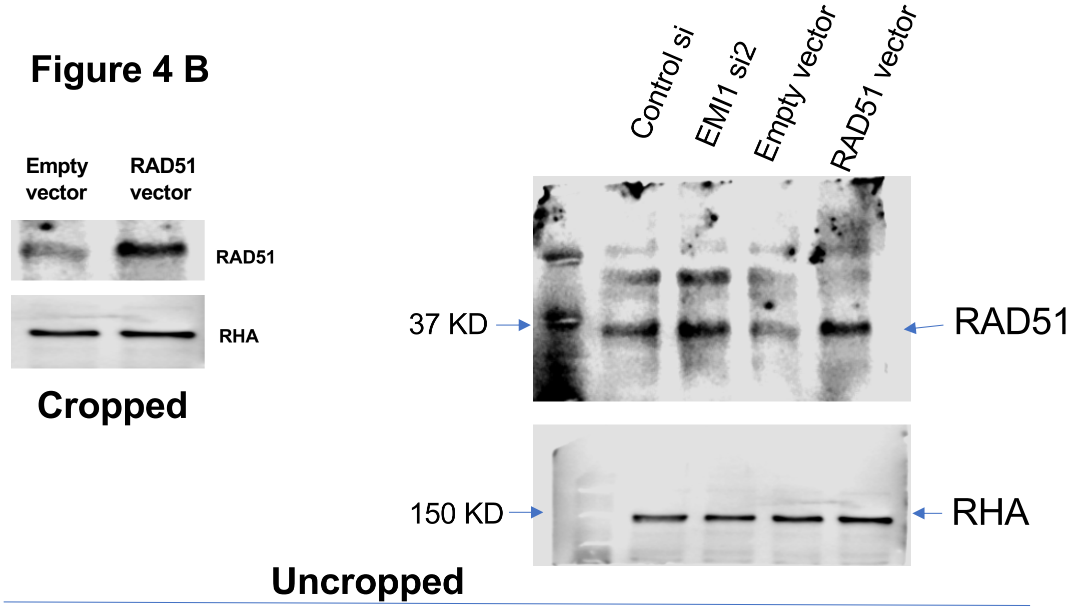

Figure 4 C

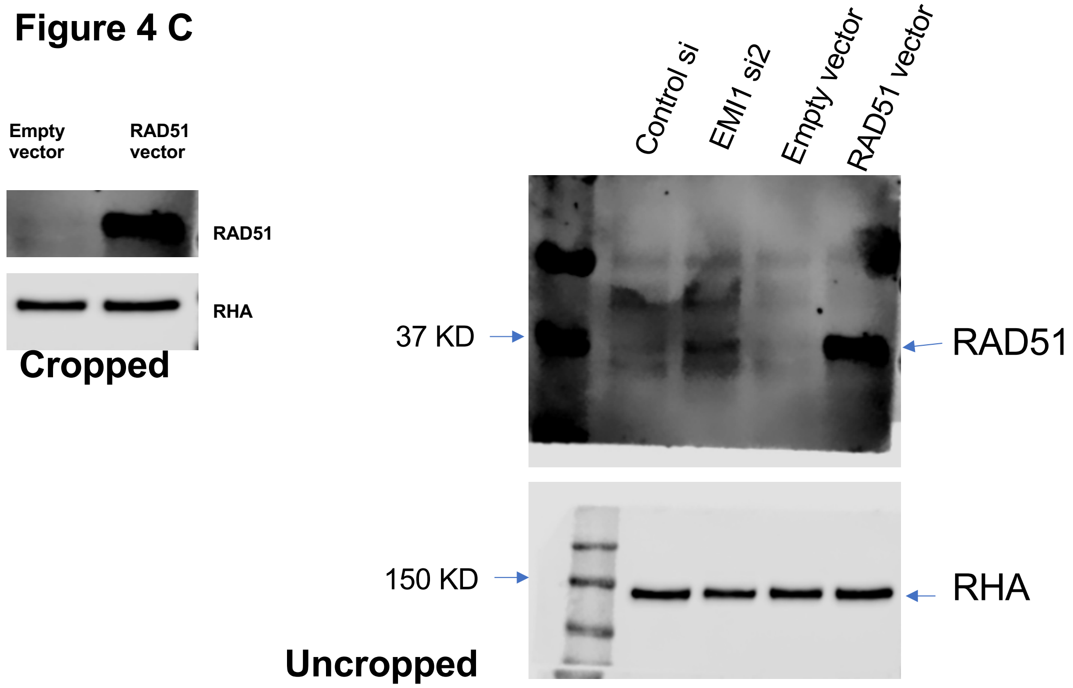

Figure 5 A

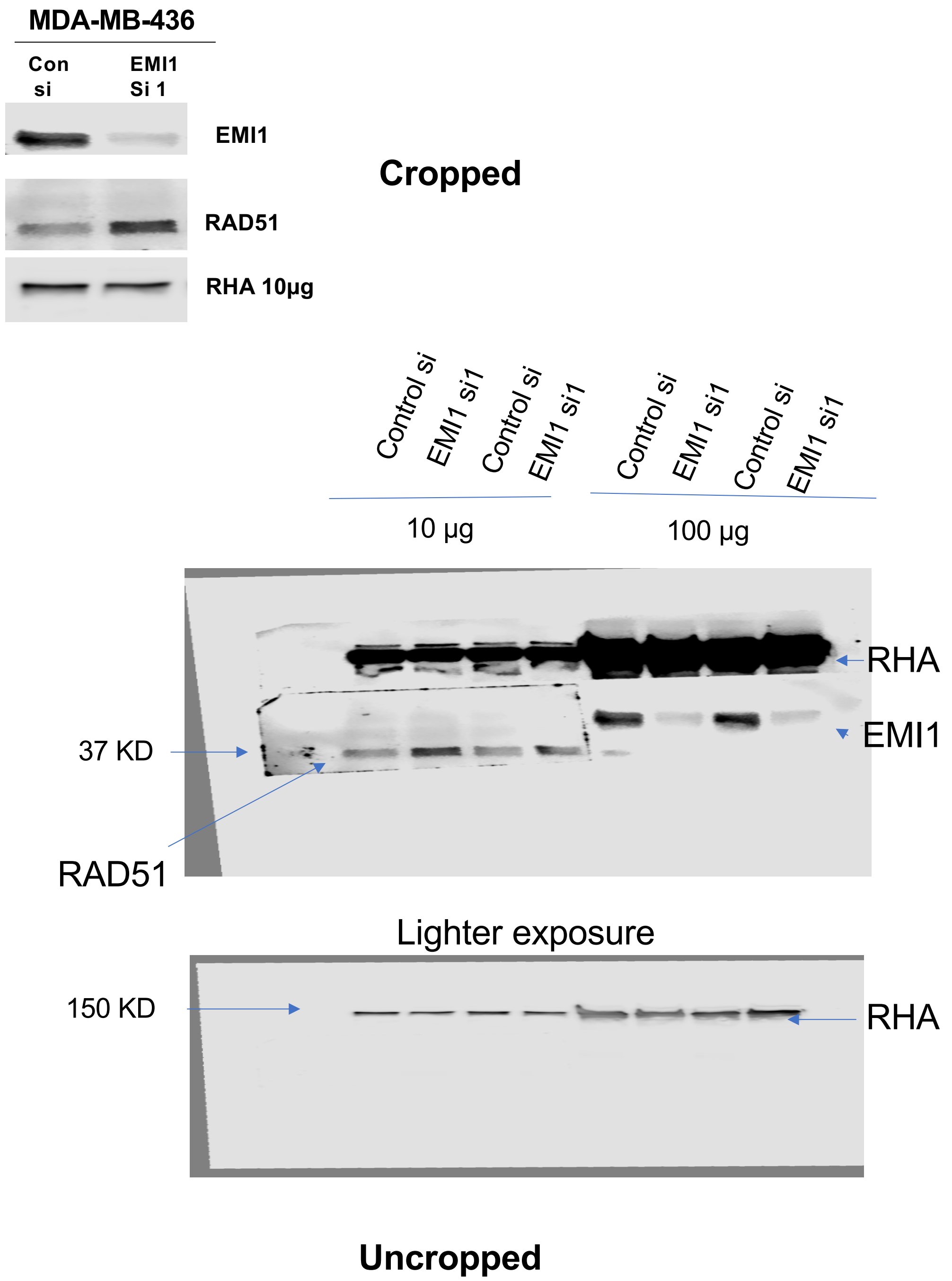
MDA-MB-436
Con si
EMI1 Si 1
EMI1
RAD51
RHA 10µg
Cropped
Control si
EMI1 si1
Control si
EMI1 si1
Control si
EMI1 si1
Control si
EMI1 si1
10 µg
100 µg
RHA
EMI1
37 KD
RAD51
Lighter exposure
150 KD
RHA
Uncropped

Figure 5 B

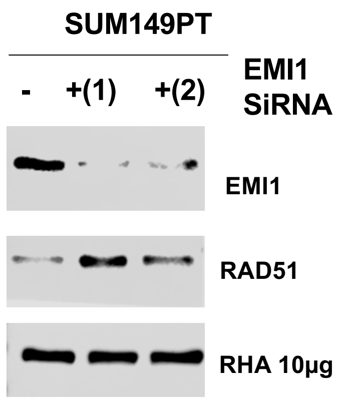

Cropped

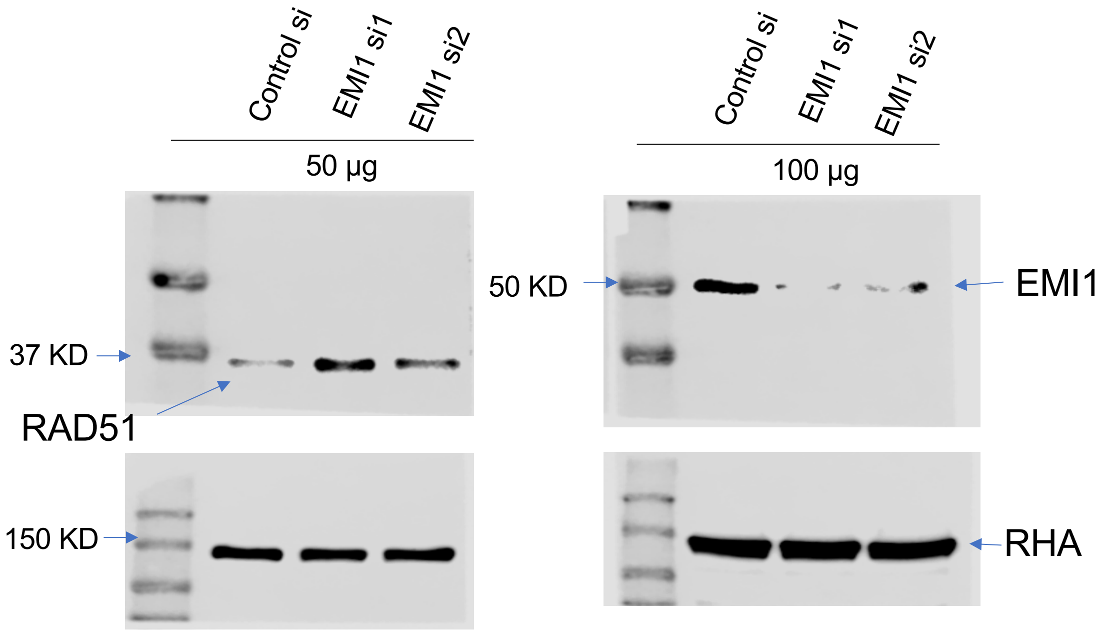

Uncropped
